# Departure Process of Actively Managed Queue with Dependent Job Sizes

**DOI:** 10.3390/e28010093

**Published:** 2026-01-13

**Authors:** Andrzej Chydzinski

**Affiliations:** Department of Computer Networks and Systems, Silesian University of Technology, Akademicka 16, 44-100 Gliwice, Poland; andrzej.chydzinski@polsl.pl

**Keywords:** queueing model, performance evaluation, departure process, rejection process, actively managed queue, dependent job sizes, dependent service times, transient analysis, stationary analysis

## Abstract

We focus on a queueing model in which the sizes of arriving jobs are stochastically dependent and each job may be denied service with a probability determined by the queue size (active management). Both of these effects are known to occur in computer networking and many other real-world realizations of queueing systems. For such a model, we perform a thorough transient and stationary analysis of the job departure process and the job rejection process. The results include theorems on the expected number of jobs that depart within a specified time interval, the departure intensity at a given time, the stationary departure rate, the expected number of jobs rejected within a specified interval, the transient rejection intensity and the stationary rejection rate. Sample numerical calculations are provided for illustration. They include various settings of the level of dependence between jobs, job rejection probabilities, and system load, as well as their impact on the departure and rejection processes.

## 1. Introduction

A detailed description of the departure process of a queueing system constitutes a very informative and often overlooked way of describing overall system performance. Specifically, the stationary departure rate represents the system’s ability to process a given number of jobs per unit time, averaged over a long period. Therefore, it is among the crucial performance characteristics. Its transient counterpart, i.e., the time-dependent intensity of the departure process, provides nuanced insight into the short-term behaviour of the system, which is influenced by particular initial conditions, as well as insight into the convergence rate to the stationary regime, etc.

Furthermore, the characterization of the departure process becomes especially important when a queueing system does not stand alone but is connected in series to another queueing (or other type of) system. In that instance, the second system is fed by the stream of jobs departing from the first system. Hence, analysis of the performance of the second system is virtually unattainable without a thorough specification of the departure stream from the first system.

All of this is often overlooked in the sense that, in many textbooks on queueing theory, there is no section devoted to an analysis of the departure process. Among scientific papers, those devoted to the departure process are much rarer than those devoted to classic performance measures of queueing models, such as queue size or waiting time.

In this paper, a thorough analysis of the departure process of a very general queueing model is performed. The model is general for the following three reasons. First, it incorporates active management—a scheme in which each arriving job may be denied service (rejected) with a probability determined by the queue size. Such active management is parameterized by function d(i), which assigns the probability of rejection to the size of the queue, *i*. In this paper, we assume a general form of d(i). This automatically incorporates classic (passive) queue management, i.e., with zero rejection probability for every *i*.

Second, the model incorporates possible stochastic dependence in the sizes of arriving jobs, resulting in dependent service times. In particular, arbitrary correlation magnitude between successive service times can be modelled. Again, when this correlation is zero, we are back in the classic case.

Third, the interarrival time distribution is arbitrary in this model. Hence, any distribution can be substituted in particular applications, including light-tailed, heavy-tailed, discrete, continuous, mixed, and others.

The assumed features of the model are not artificial. In fact, they are known to occur in many different real-world realizations of queueing systems. Rejections occurring with a probability determined by the queue size represent a natural phenomenon when the queue consists of human customers. Namely, people often decide not to join a queue when it is too long. The particular decision is inherently stochastic—it depends on the personal patience of each customer, his or her need for the service, and other random factors.

Exactly the same scheme has been proposed for the Internet, i.e., deleting packets arriving at a networking device with a probability determined by the number of packets already waiting for transmission. This mechanism is intended to mitigate several problems caused by bufferbloat—frequent overflowing of the entire buffering space in networking devices.

Similarly, the stochastic dependence of job sizes and service times occur in both human queues and computer networking. Specifically, the dependence of service times in human queues can be caused by external events. A natural disaster may cause many patients to arrive at the emergency room, needing similar assistance. A failure of the electricity grid in part of a city may induce many phone calls to a company hotline, with similar needs for information. In computer networking, on the other hand, stochastically dependent service times of packets arise directly from their dependent sizes, caused by networking protocols.

For all these reasons, in this paper, a thorough analysis of the departure process of an actively managed queue with dependent job sizes is performed. We begin with the transient analysis. Specifically, a formula for the expected number of jobs departing within interval (0,t) is derived first. From this result, we obtain the intensity of the departure process at arbitrary *t*. This characteristic provides a full and intuitive description of the evolution of the departure process over the entire time axis, t∈(0,∞). After that, it is straightforward to obtain the stationary departure rate as the limit of the time-dependent intensity as t→∞.

In addition to the analysis of the departure process, a complementary analysis of the rejection process is performed. This consists of deriving the expected number of jobs rejected within (0,t), the rejection intensity at arbitrary *t*, and the stationary rejection rate.

The theoretical results are supported by sample numerical calculations. These are performed for various settings regarding the strength of the dependence between service times, job rejection probabilities, and system load. They demonstrate the impact of these contributors on the departure and rejection processes.

In the model, the correlated service times are imitated by the Markovian service process of arbitrary parameter matrices. This process enables the modeling of an arbitrary strength correlation between service times. This will be illustrated in numerical examples, where cases ranging from a very weak correlation up to a strong correlation will be demonstrated.

Summarizing, the detailed contributions of this work are as follows:

- Theorem on expected number of jobs departed in (0,t)—Theorem 1;
- Theorem on intensity of departure process at arbitrary *t*—Corollary 1;
- Theorem on stationary departure rate—Corollary 2;
- Theorem on expected number of jobs rejected in (0,t)—Theorem 2;
- Theorem on intensity of rejection process at arbitrary *t*—Corollary 3;
- Theorem on stationary rejection rate—Corollary 4;
- Numeric examples, demonstrating the impact of correlation, rejection probabilities and system load on departure and rejection processes.

The paper’s remaining content is organized as described next. In Section 2, the relevant previous studies are outlined. In Section 3, the queueing model under study is described thoroughly. Then, Section 4 and Section 5 constitute pivotal parts of the paper. Specifically, Section 4 is focused on an analysis of the departure process. A detailed characterization of this process is presented in Theorem 1, Corollaries 2 and 3 in this section. Similarly, Section 5 deals with the rejection process. Its main contributions are Theorem 2, and Corollaries 3 and 4, on the characteristics of this process. Then, in Section 6, the sample calculation results are presented. This section is segmented into four parts, numbered Section 6.1, Section 6.2, Section 6.3 and Section 6.4. Specifically, in Section 6.1, the impact of rejection probabilities on the departure process is illustrated. Four different functions d(i) are employed in these examples. In Section 6.2, the influence of correlation on the departure process is shown. Various correlation strengths are considered, from a very weak (R=0.05), to a strong one (R=0.5). In Section 6.3, the impact of the load is depicted, using load values from a deep underload (ρ=0.25) up to a strong overload (ρ=1.5). Section 6.4 presents a comparison of the simulation results, obtained using OMNeT++, with the theoretical results of the paper. This is meant to ensure that the derivations are error-free. Finally, Section 7 brings together the paper’s concluding remarks.

## 2. Related Work

Based on the author’s awareness, no previous mathematical study dealt with the departure or rejection process of a queueing model in which each arriving job may be randomly rejected basing on the queue length and service times are stochastically dependent.

The impact of each of these phenomena has been studied separately rather than in combination.

For instance, in [1,2,3,4,5,6,7,8,9], models with dependent service times were investigated. Various performance characteristics were studied, including queue size [1,2,3,5,9], waiting time [1,4,6,7], idle period [5], busy period [5,7], and others. Furthermore, various versions of the queueing model were considered, including an infinite waiting room in [1,2,3,4,5,6,9], a finite waiting room in [1,7,8], group arrivals in [5,6,7], and group service in [2,8,9].

In none of the models of [1,2,3,4,5,6,7,8,9], however, were probabilistic job rejections taken into account, as in the model investigated here.

On the other hand, active management based on probabilistic rejections has been studied extensively in computer networking. Various forms of job/packet rejection probabilities d(i) have been proposed, e.g., linear [10], trigonometric [11], polynomial [12,13], Gaussian [14], exponential [15,16], beta [17], mixed [18,19,20,21], and others [22].

The majority of the papers mentioned in the preceding paragraph rely either on computer simulations or networking experiments as the main tools of performance evaluation. On the other hand, mathematical studies of such models were carried out, for instance, in [23,24,25,26,27,28,29].

Furthermore, probabilistic job/packet rejection schemes may include other mechanisms, in which the rejection probability is not a direct function of the queue size (see, e.g., [30,31,32,33,34,35,36,37]).

None of the mentioned models of active management, [10,11,12,13,14,16,17,18,19,20,21,23,24,25,26,27,28,29,30,31,32,33,34,35,36,37], incorporate a mathematical analysis of the impact of stochastically dependent service times, as is carried out here.

In light of the previous work, the motivation behind this paper is twofold. First, a very general queueing model is analyzed, capable of simultaneously mimicking two important phenomena occurring in queues of human customers and electronic devices. Second, the paper provides a detailed characterization of the departure process, which is a very informative, although somewhat underappreciated, way of describing overall system performance.

## 3. Queue Model

We analyze a single-server queue, such that the job arrival stream is a general renewal process, i.e., has mutually independent interarrival times, which are distributed according to distribution function G(t), of arbitrary form.

Jobs generate a queue in the waiting room, which is served/drained from the head. Upon arrival in the queue, each job can be rejected randomly, with probability d(i), where *i* stands for the queue size upon the arrival of this job. A rejected job cannot join the queue—it leaves the queue and never returns. The waiting room (buffer), in which the queue is formed, is finite and has a capacity of *N* jobs (which includes service position). An arriving job that finds the waiting room is full must be rejected. This is equivalent of setting d(i)=1 for i≥N. In the range 0≤i<N, function d(i) can assume any values in interval [0,1).

The sizes of consecutive jobs are stochastically dependent, which makes the service times stochastically dependent. Specifically, the service times and their dependence are represented by the Markovian service process, MSP, [1]. This process is parameterized by two M×M matrices: $L_{0}$ and $L_{1}$. $L_{0}$ must be negative on the diagonal, while otherwise non-negative; $L_{1}$ must be non-negative. Furthermore, $L=L_{0}+L_{1}$ must constitute a rate matrix for a Markov chain with space of states {1,…,M}. These states will be called “service states”.

The detailed evolution of an MSP is as follows. If there is a job under service at time *t*, while the service state is *j*, then right after, at t+Δ, the service state may switch to *k* with probability ${\left(L_{0}\right)}_{jk}\Delta +o\left(\Delta \right)$ and the service of the job is continued, or the service state may switch to *k* with the probability ${\left(L_{1}\right)}_{jk}\Delta +o\left(\Delta \right)$ and the service of the job is completed, so the job leaves the queue.

When the queue becomes empty after a job departure, the service state remains the same for the whole idle time. After that time, when a new job arrives in the system, the service state resumes its evolution, as described in the previous paragraph.

Let S(t) denote the service state at *t*. Note that S(t) is not a Markov chain. Specifically, in a busy period, S(t) evolves in Markovian manner, but this behaviour is disturbed during the successive idle period. Therefore, as a whole, S(t) is not Markovian.

The service rate, μ, is calculated as follows:

(1) $$\mu =\pi L_{1}e,$$

where vector π is the solution of the system:

(2) $$\begin{array}{c} \left\{\begin{array}{c} \pi L=[0,\ldots ,0],\\ \pi e=1, \end{array}\right. \end{array}$$

while **e** is a column vector of 1’s.

Two very important parameters of the system, which severely affect its performance, are ρ and *R*. Namely, using ρ, we denote the system load, while *R* represents the correlation coefficient between successive service times.

The two parameters can be calculated as follows:

(3) $$\rho ={\left(\mu \int_{0}^{\infty }tdG\left(t\right)\right)}^{-1},$$

and

(4) $$R=\frac{\mu \pi L^{-}L_{1}L^{-}e-1}{2\mu \pi L^{-}e-1},\;\;\;\;\;\;L^{-}=-L_{0}^{-1},$$

(see ref. [38] for proof of the latter).

Hereafter X(t) stands for the queue size at *t*, which includes the position for the service if busy at *t*. Similarly, argument *i* of function d(i) is assumed to incorporate the position for the service, if busy. Moreover, if *t* is a time of successful (not rejected) arrival, then it is assumed that X(t) includes the job that just arrived and joined the queue. This assumption makes X(t) right continuous, i.e., X(0+)=X(t) for every *t*.

Lastly, we assume that t=0 is an arrival epoch if X(0)>0, i.e., the earliest job arrives after a time distributed according to G(t). Similarly, when X(0)=0, the earliest arrival happens at a time distributed according to G(t).

## 4. Departure Process

Let $C_{m,j}\left(t\right)$ denote the expected number of jobs completed and departed in interval (0,t), given that X(0)=m, S(0)=j. Then, the intensity of the departure process is

(5) $$C_{m,j}^{\prime }\left(t\right)=\frac{dC_{m,j}\left(t\right)}{dt},$$

while the stationary departure rate is

(6) $$C=\lim_{t\to \infty }C_{0,1}^{\prime }\left(t\right).$$

We begin derivations with $C_{m,j}\left(t\right)$. After that, it will be easy to obtain $C_{m,j}^{\prime }\left(t\right)$ and *C*.

We can first construct an integral equation for $C_{0,j}\left(t\right)$, assuming that there are no jobs in the system at time origin. We have

(7) $$\begin{array}{cc} C_{0,j}\left(t\right)= & d\left(0\right)\int_{0}^{t}C_{0,j}\left(t-u\right)dG\left(u\right)+\left(1-d(0)\right)\int_{0}^{t}C_{1,j}\left(t-u\right)dG\left(u\right). \end{array}$$

The latter equation can be acquired by conditioning on the earliest arrival time, together with rejection or acceptance of the first job. Specifically, with probability dG(u)d(0), a job arrives at u<t and is rejected. In such cases, the queue size is always 0 at time *u*, and the service state is still *j*. Hence, the expected number of jobs departed by *t* becomes $C_{0,j}\left(t-u\right)$. Similarly, with probability dG(u)(1−d(0)) a job arrives at u<t and is accepted. In such cases, the queue size is 1 at time *u*, so the expected number of jobs departed by *t* is then $C_{1,j}\left(t-u\right)$. If the earliest arrival happens after *t*, then the expected number of jobs departed by *t* is 0. Therefore, there is no corresponding summand in (7).

Now, we can construct an equation for $C_{N,j}\left(t\right)$, i.e., assuming the waiting room is saturated at the origin time. We obtain

(8) $$\begin{array}{cc} C_{N,j}\left(t\right)= & \sum_{i=0}^{N-1}\sum_{k=1}^{M}d\left(N-i\right)\int_{0}^{t}q_{jk}\left(u,i\right)\left(i+C_{N-i,k}\left(t-u\right)\right)dG\left(u\right)\\ \; & +\sum_{i=1}^{N-1}\sum_{k=1}^{M}\left(1-d(N-i)\right)\int_{0}^{t}q_{jk}\left(u,i\right)\left(i+C_{N-i+1,k}\left(t-u\right)\right)dG\left(u\right)\\ \; & +\sum_{k=1}^{M}d\left(0\right)\int_{0}^{t}r_{jk}\left(u,N\right)\left(N+C_{0,k}\left(t-u\right)\right)dG\left(u\right)\\ \; & +\sum_{k=1}^{M}\left(1-d(0)\right)\int_{0}^{t}r_{jk}\left(u,N\right)\left(N+C_{1,k}\left(t-u\right)\right)dG\left(u\right)\\ \; & +\sum_{i=0}^{N}\sum_{k=1}^{M}iq_{jk}\left(t,i\right)\left(1-G(t)\right), \end{array}$$

where function $q_{jk}\left(u,l\right)$ expresses the probability that *l* jobs depart in an interval of length *u* and the service state is *k* at the end of this interval, assuming that, at the opening of the interval, the queue size is *l* and the service state is *j*. Function $r_{jk}\left(u,l\right)$ expresses the probability that *l* jobs depart in an interval of length *u* and the service state is *k* at the end of the *l*-th service, assuming that, at the opening of the interval, the queue size is *l* and the service state is *j*.

Equation (8) can be acquired by conditioning on the earliest arrival time, together with rejection or acceptance of the first job, with the number of jobs that have departed by the earliest arrival time, and with the service state at that time. Specifically, with probability $dG\left(u\right)q_{jk}\left(u,i\right)d\left(N-i\right)$, a job arrives at u<t, *i* other jobs depart by *u*, the job arriving at *u* is rejected, and the service state at *u* is *k*. In such cases, the queue length is N−i at time *u* and the expected number of jobs that will depart by *t* becomes $i+C_{N-i,k}\left(t-u\right)$, which is presented in the first summand of (8). The second summand addresses the situation where the first job is accepted. This, however, can only happen if at least one job departs by time *u*, thus freeing some space in the waiting room. Hence, the sum $\sum_{i=1}^{N-1}$ must begin with i=1. The third summand of (8) addresses the situation where the earliest arrival is rejected, but by the time of the earliest arrival, *N* jobs have already departed from the system, meaning the system is empty. In such cases, the expected number of jobs that depart by *t* becomes $N+C_{0,k}\left(t-u\right)$. The fourth summand is like the third one, except that the earliest arrival is accepted, which makes the expected number of jobs that departed by *t* $N+C_{1,k}\left(t-u\right)$. Finally, the last summand addresses the case where the earliest arrival is after *t*. Hence, the number of jobs that departed by *t* is *i*, with probability $q_{jk}\left(t,i\right)$.

Then, we can construct an equation for $C_{m,j}\left(t\right)$, assuming that, at the origin time, the waiting room is not empty or full. Specifically, for 0<m<N we have

(9) $$\begin{array}{cc} C_{m,j}\left(t\right)=\; & \sum_{i=0}^{m-1}\sum_{k=1}^{M}d\left(m-i\right)\int_{0}^{t}q_{jk}\left(u,i\right)\left(i+C_{m-i,k}\left(t-u\right)\right)dG\left(u\right)\\ \; & +\sum_{i=0}^{m-1}\sum_{k=1}^{M}\left(1-d(m-i)\right)\int_{0}^{t}q_{jk}\left(u,i\right)\left(i+C_{m-i+1,k}\left(t-u\right)\right)dG\left(u\right)\\ \; & +\sum_{k=1}^{M}d\left(0\right)\int_{0}^{t}r_{jk}\left(u,m\right)\left(m+C_{0,k}\left(t-u\right)\right)dG\left(u\right)\\ \; & +\sum_{k=1}^{M}\left(1-d(0)\right)\int_{0}^{t}r_{jk}\left(u,m\right)\left(m+C_{1,k}\left(t-u\right)\right)dG\left(u\right)\\ \; & +\sum_{i=0}^{m}\sum_{k=1}^{M}iq_{jk}\left(t,i\right)\left(1-G(t)\right). \end{array}$$

Equation (9) can be acquired in an almost identical way as (8), with one exception, i.e., the second summand of (9). This addresses the case where the first job is accepted. Because the waiting room is not initially saturated, the situation where no jobs departed by time *u* is allowed in this situation. Hence, the second summand must now begin with i=0, rather than i=1, as in (8).

We will exploit the Laplace transform for the purpose of solving system (7)–(9) as follows:

(10) $$c_{m,j}\left(s\right)=\int_{0}^{\infty }e^{-st}C_{m,j}\left(t\right)dt,$$

(11) $$G^{*}\left(s\right)=\int_{0}^{\infty }e^{-st}dG\left(t\right),$$

(12) $$a_{jk}\left(s,l\right)=\int_{0}^{\infty }e^{-st}q_{jk}\left(t,l\right)dG\left(t\right),$$

(13) $$h_{jk}\left(s,l\right)=\int_{0}^{\infty }e^{-st}r_{jk}\left(t,l\right)dG\left(t\right),$$

(14) $$p_{jk}\left(s,l\right)=\int_{0}^{\infty }e^{-st}q_{jk}\left(t,l\right)\left(1-G(t)\right)dt.$$

Applying the theorem on the transform of the convolution (see, e.g., p. 92 of [39]), Equation (7) yields

(15) $$\begin{array}{cc} c_{0,j}\left(s\right)= & d\left(0\right)G^{*}\left(s\right)c_{0,j}\left(s\right)+\left(1-d(0)\right)G^{*}\left(s\right)c_{1,j}\left(s\right), \end{array}$$

while from (8), we obtain

(16) $$\begin{array}{cc} c_{N,j}\left(s\right)=\; & \sum_{i=0}^{N-1}d\left(N-i\right)\sum_{k=1}^{M}a_{jk}\left(s,i\right)\left(\frac{i}{s}+c_{N-i,k}\left(s\right)\right)\\ \; & +\sum_{i=1}^{N-1}\left(1-d(N-i)\right)\sum_{k=1}^{M}a_{jk}\left(s,i\right)\left(\frac{i}{s}+c_{N-i+1,k}\left(s\right)\right)\\ \; & +d\left(0\right)\sum_{k=1}^{M}h_{jk}\left(s,N\right)\left(\frac{N}{s}+c_{0,k}\left(s\right)\right)+\left(1-d(0)\right)\sum_{k=1}^{M}h_{jk}\left(s,N\right)\left(\frac{N}{s}+c_{1,k}\left(s\right)\right)\\ \; & +\sum_{i=0}^{N}\sum_{k=1}^{M}ip_{jk}\left(s,i\right). \end{array}$$

From (9), for 0<m<N, we obtain

(17) $$\begin{array}{cc} c_{m,j}\left(s\right)=\; & \sum_{i=0}^{m-1}d\left(m-i\right)\sum_{k=1}^{M}a_{jk}\left(s,i\right)\left(\frac{i}{s}+c_{m-i,k}\left(s\right)\right)\\ \; & +\sum_{i=0}^{m-1}\left(1-d(m-i)\right)\sum_{k=1}^{M}a_{jk}\left(s,i\right)\left(\frac{i}{s}+c_{m-i+1,k}\left(s\right)\right)\\ \; & +d\left(0\right)\sum_{k=1}^{M}h_{jk}\left(s,m\right)\left(\frac{m}{s}+c_{0,k}\left(s\right)\right)+\left(1-d(0)\right)\sum_{k=1}^{M}h_{jk}\left(s,m\right)\left(\frac{m}{s}+c_{1,k}\left(s\right)\right)\\ \; & +\sum_{i=0}^{m}\sum_{k=1}^{M}ip_{jk}\left(s,i\right). \end{array}$$

It is convenient to use vectors and matrices for systems (15)–(17). Specifically, the following notations are used:

(18) $$c_{m}\left(s\right)={\left[c_{m,1}\left(s\right),\ldots ,c_{m,M}\left(s\right)\right]}^{T},$$

(19) $$A\left(s,l\right)={\left[a_{jk}\left(s,l\right)\right]}_{j=1,\ldots ,M;k=1,\ldots ,M},$$

(20) $$H\left(s,l\right)={\left[h_{jk}\left(s,l\right)\right]}_{j=1,\ldots ,M;k=1,\ldots ,M},$$

(21) $$P\left(s,l\right)={\left[p_{jk}\left(s,l\right)\right]}_{j=1,\ldots ,M;k=1,\ldots ,M}.$$

From (15), we obtain

(22) $$\begin{array}{cc} c_{0}\left(s\right)= & d\left(0\right)G^{*}\left(s\right)c_{0}\left(s\right)+\left(1-d(0)\right)G^{*}\left(s\right)c_{1}\left(s\right), \end{array}$$

whereas from (16), we obtain

(23) $$\begin{array}{cc} c_{N}\left(s\right)=\; & \sum_{i=0}^{N-1}d\left(N-i\right)A\left(s,i\right)c_{N-i}\left(s\right)\\ \; & +\sum_{i=1}^{N-1}\left(1-d(N-i)\right)A\left(s,i\right)c_{N-i+1}\left(s\right)\\ \; & +d\left(0\right)H\left(s,N\right)c_{0}\left(s\right)+\left(1-d(0)\right)H\left(s,N\right)c_{1}\left(s\right)+y_{N}\left(s\right), \end{array}$$

with

(24) $$y_{m}\left(s\right)=\frac{1}{s}\left(\sum_{i=0}^{m-1}iA\left(s,i\right)+mH\left(s,m\right)+\sum_{i=0}^{m}siP\left(s,i\right)\right)e.$$

Similarly, (17) yields for 0<m<N:

(25) $$\begin{array}{cc} c_{m}\left(s\right)=\; & \sum_{i=0}^{m-1}d\left(m-i\right)A\left(s,i\right)c_{m-i}\left(s\right)\\ \; & +\sum_{i=0}^{m-1}\left(1-d(m-i)\right)A\left(s,i\right)c_{m-i+1}\left(s\right)\\ \; & +d\left(0\right)H\left(s,m\right)c_{0}\left(s\right)+\left(1-d(0)\right)H\left(s,m\right)c_{1}\left(s\right)+y_{m}\left(s\right). \end{array}$$

As we see, (22), (23) and (25) now constitute a system of linear equations with matrix coefficients. Therefore, its solution is easy to acquire using the standard algebra, and may be presented in the form of a vector:

(26) $$c\left(s\right)={\left[c_{0}{\left(s\right)}^{T},c_{1}{\left(s\right)}^{T},\ldots ,c_{N}{\left(s\right)}^{T}\right]}^{T}.$$

Namely, grouping all $c_{m}\left(s\right)$ variables on one side and known coefficients on the other in Equations (22), (23) and (25), we acquire the outcome as follows.

**Theorem 1.**  *The expected number of jobs departed from the queue in (0,t) has the following transform:*

(27) $$c\left(s\right)=F^{-1}\left(s\right)z\left(s\right),$$

*where*

(28) $$F\left(s\right)={\left[F_{m,k}\left(s\right)\right]}_{m=0,\ldots ,N;k=0,\ldots ,N},$$

(29) $$\begin{array}{cc} \; & F_{m,k}\left(s\right)=\\ \; & =\left\{\begin{array}{cc} G^{*}\left(s\right)d\left(0\right)I-I, & \mathrm{if}\;\;m=k=0,\\ G^{*}\left(s\right)\left(1-d(0)\right)I, & \mathrm{if}\;\;m=0,k=1,\\ d\left(1\right)A\left(s,m-1\right)+\left(1-d(0)\right)H\left(s,m\right)-I, & \mathrm{if}\;\;m=k=1,\\ d(0)H(s,m), & \mathrm{if}\;\;1\leq m\leq N,k=0,\\ d\left(1\right)A\left(s,m-1\right)+\left(1-d(0)\right)H\left(s,m\right), & \mathrm{if}\;\;2\leq m\leq N,k=1,\\ \left(1-d(m)\right)A\left(s,0\right), & \mathrm{if}\;\;1\leq m\leq N-1,k=m\;+\;1,\\ d\left(k\right)A\left(s,m-k\right)+\left(1-d(k-1)\right)A\left(s,m-k+1\right)-I, & \mathrm{if}\;\;2\leq m\leq N,k=m,\\ d\left(k\right)A\left(s,m-k\right)+\left(1-d(k-1)\right)A\left(s,m-k+1\right), & \mathrm{if}\;\;2\leq m\leq N,2\leq k\leq m\;-\;1,\\ 0, & otherwise, \end{array}\right. \end{array}$$

*and*

(30) $$z\left(s\right)=-{\left[0^{T},y_{1}{\left(s\right)}^{T},\ldots ,y_{N}{\left(s\right)}^{T}\right]}^{T},$$

$y_{m}\left(s\right)$ *is given in (24), and 0 denotes the M×M zero matrix, whereas **I** denotes the identity matrix of size M×M.*

From Theorem 1, it is easy to obtain the intensity of the departure process, defined in (5), and the stationary departure rate, (6). Specifically, denoting

(31) $$c_{m,j}^{*}\left(s\right)=\int_{0}^{\infty }e^{-st}C_{m,j}^{\prime }\left(t\right)dt,$$

after applying the derivative theorem (p. 54 of [39]), to (27) and (5), we obtain the corollary as follows.

**Corollary 1.**  *The intensity of the departure process at t has the following transform:*

(32) $$c_{m,j}^{*}\left(s\right)=s{\left[F^{-1}\left(s\right)z\left(s\right)\right]}_{mM+j},$$

*where F(s) and z(s) are presented in Theorem 1, while ${\left[\cdot \right]}_{k}$ denotes the k-th entry of a vector.*

To obtain *C*, we can administer the final-value theorem (p. 89 of [39]) to (32) and (6). This leads to the following outcome.

**Corollary 2.**  *The stationary departure rate is*

(33) $$C=\lim_{s\to 0+}s^{2}{\left[F^{-1}\left(s\right)z\left(s\right)\right]}_{1}.$$

To employ Theorem 1 and Corollaries 1 and 2 in practice, we are in need of coefficients A(s,m), H(s,m), occurring in (29), and a method for inversion of the transform (Theorem 1 and Corollary 1, only). Coefficients A(s,m), H(s,m) were computed in Section 5 of [40]. To invert the Laplace transform, the method of [41] is used in Section 6.

This completes the analysis of the departure process from both the theoretical and computational perspectives.

## 5. Rejection Process

Let $L_{m,j}\left(t\right)$ denote the expected number of jobs rejected in interval (0,t), given that X(0)=m, S(0)=j. Then, the intensity of the rejection process is

(34) $$L_{m,j}^{\prime }\left(t\right)=\frac{dC_{m,j}\left(t\right)}{dt},$$

while the stationary rejection rate is

(35) $$L=\lim_{t\to \infty }L_{0,1}^{\prime }\left(t\right).$$

We can first design an equation for $L_{0,j}\left(t\right)$, assuming X(0)=0. We have

(36) $$\begin{array}{c} L_{0,j}\left(t\right)=d\left(0\right)\int_{0}^{t}\left(1+L_{0,j}\left(t-u\right)\right)dG\left(u\right)+\left(1-d(0)\right)\int_{0}^{t}L_{1,j}\left(t-u\right)dG\left(u\right). \end{array}$$

Namely, with probability dG(u)d(0) a job arrives at u<t and is rejected. Hence, the expected number of jobs rejected by *t* becomes $1+L_{0,j}\left(t-u\right)$ at *u*, as neither the queue size nor the service state change at *u*. With probability dG(u)(1−d(0)), a job arrives at u<t and is accepted. In such cases, the expected number of jobs rejected by *t* is $L_{1,j}\left(t-u\right)$ at *u*, as the queue length grows to 1 at *u*. If the earliest arrival happens after *t*, then the expected number of jobs rejected by *t* must be 0.

Second, we can design an equation for $L_{N,j}\left(t\right)$, i.e., assuming X(0)=N. We obtain the following:

(37) $$\begin{array}{cc} L_{N,j}\left(t\right)= & \sum_{i=0}^{N-1}\sum_{k=1}^{M}d\left(N-i\right)\int_{0}^{t}q_{jk}\left(u,i\right)\left(1+L_{N-i,k}\left(t-u\right)\right)dG\left(u\right)\\ \; & +\sum_{i=1}^{N-1}\sum_{k=1}^{M}\left(1-d(N-i)\right)\int_{0}^{t}q_{jk}\left(u,i\right)L_{N-i+1,k}\left(t-u\right)dG\left(u\right)\\ \; & +\sum_{k=1}^{M}d\left(0\right)\int_{0}^{t}r_{jk}\left(u,N\right)\left(1+L_{0,k}\left(t-u\right)\right)dG\left(u\right)\\ \; & +\sum_{k=1}^{M}\left(1-d(0)\right)\int_{0}^{t}r_{jk}\left(u,N\right)L_{1,k}\left(t-u\right)dG\left(u\right). \end{array}$$

As for the first summand of (37), with probability $dG\left(u\right)q_{jk}\left(u,i\right)d\left(N-i\right)$ a job arrives at *u*, *i* jobs are departed by *u*, the job arriving at *u* is rejected, and the service state at *u* is *k*. In such cases, the expected number of jobs rejected by *t* becomes $1+L_{N-i,k}\left(t-u\right)$, as the queue size changes to N−i at *u*. Regarding the second summand, the first job is accepted at *u* and the queue size becomes N−i+1, so the expected number of jobs rejected by *t* becomes $L_{N-i+1,k}\left(t-u\right)$. The third and fourth summand address the case where the system is empty by *u*. Therefore, at *u*, the expected number of jobs rejected by *t* becomes either $1+L_{0,k}$, if the first job is rejected (third summand), or $L_{1,k}\left(t-u\right)$, if the first job is accepted (fourth summand). Note that if the earliest arrival is after *t*, there cannot be any job rejections by *t* (a job can be rejected only on arrival).

Then, we can design an equation for $L_{m,j}\left(t\right)$ assuming 0<X(0)<N. We have

(38) $$\begin{array}{cc} L_{m,j}\left(t\right)=\; & \sum_{i=0}^{m-1}\sum_{k=1}^{M}d\left(m-i\right)\int_{0}^{t}q_{jk}\left(u,i\right)\left(1+L_{m-i,k}\left(t-u\right)\right)dG\left(u\right)\\ \; & +\sum_{i=0}^{m-1}\sum_{k=1}^{M}\left(1-d(m-i)\right)\int_{0}^{t}q_{jk}\left(u,i\right)L_{m-i+1,k}\left(t-u\right)dG\left(u\right)\\ \; & +\sum_{k=1}^{M}d\left(0\right)\int_{0}^{t}r_{jk}\left(u,m\right)\left(1+L_{0,k}\left(t-u\right)\right)dG\left(u\right)\\ \; & +\sum_{k=1}^{M}\left(1-d(0)\right)\int_{0}^{t}r_{jk}\left(u,m\right)L_{1,k}\left(t-u\right)dG\left(u\right). \end{array}$$

Equation (38) differs from (37) only in the second summand. Namely, in (37) it is not allowed for a new job to join the queue when there are no departures by *u*, as the waiting room is saturated at *u*. Thus, i=0 is excluded in the second summand of (37), whereas it is not excluded in (38), in which the waiting room is not saturated at *u*.

Denoting

(39) $$l_{m,j}\left(s\right)=\int_{0}^{\infty }e^{-st}L_{m,j}\left(t\right)dt,$$

(40) $$l_{m}\left(s\right)={\left[l_{m,1}\left(s\right),\ldots ,l_{m,M}\left(s\right)\right]}^{T},$$

we can proceed with (36)–(38) in an analogous manner, as with (7)–(9) in Section 4, respectively.

After that, Equation (36) becomes

(41) $$\begin{array}{cc} l_{0}\left(s\right)= & d\left(0\right)G^{*}\left(s\right)l_{0}\left(s\right)+\left(1-d(0)\right)G^{*}\left(s\right)l_{1}\left(s\right)+u\left(s\right), \end{array}$$

where

(42) $$u\left(s\right)=\frac{d\left(0\right)G^{*}\left(s\right)}{s}e.$$

Equation (37) leads to

(43) $$\begin{array}{cc} l_{N}\left(s\right)=\; & \sum_{i=0}^{N-1}d\left(N-i\right)A\left(s,i\right)l_{N-i}\left(s\right)\\ \; & +\sum_{i=1}^{N-1}\left(1-d(N-i)\right)A\left(s,i\right)l_{N-i+1}\left(s\right)\\ \; & +d\left(0\right)H\left(s,N\right)l_{0}\left(s\right)+\left(1-d(0)\right)H\left(s,N\right)l_{1}\left(s\right)+x_{N}\left(s\right), \end{array}$$

with

(44) $$x_{m}\left(s\right)=\frac{1}{s}\left(\sum_{i=0}^{m-1}d\left(m-i\right)A\left(s,i\right)+d\left(0\right)H\left(s,m\right)\right)e,$$

and (38) leads to

(45) $$\begin{array}{cc} l_{m}\left(s\right)=\; & \sum_{i=0}^{m-1}d\left(m-i\right)A\left(s,i\right)l_{m-i}\left(s\right)\\ \; & +\sum_{i=0}^{m-1}\left(1-d(m-i)\right)A\left(s,i\right)l_{m-i+1}\left(s\right)\\ \; & +d\left(0\right)H\left(s,m\right)l_{0}\left(s\right)+\left(1-d(0)\right)H\left(s,m\right)l_{1}\left(s\right)+x_{m}\left(s\right),\;\;\;0<m<N. \end{array}$$

As previously, (41), (43) and (45) constitute a system od linear equations with matrix coefficients. Its solution can be acquired with standard algebra. Denoting

(46) $$l\left(s\right)={\left[l_{0}{\left(s\right)}^{T},l_{1}{\left(s\right)}^{T},\ldots ,l_{N}{\left(s\right)}^{T}\right]}^{T},$$

we have the following results.

**Theorem 2.**  *The expected number of rejected jobs in (0,t) has the following transform:*

(47) $$l\left(s\right)=F^{-1}\left(s\right)w\left(s\right)$$

*where*

(48) $$w\left(s\right)=-{\left[u{\left(s\right)}^{T},x_{1}{\left(s\right)}^{T},\ldots ,x_{N}{\left(s\right)}^{T}\right]}^{T},$$

*while F(s), u(s) and $x_{m}\left(s\right)$ are given in (29), (42), and (44), respectively.*

Denoting

(49) $$l_{m,j}^{*}\left(s\right)=\int_{0}^{\infty }e^{-st}L_{m,j}^{\prime }\left(t\right)dt,$$

and applying the derivative theorem to (47), we obtain the corollary as follows.

**Corollary 3.**  *The intensity of the rejection process at t has the following transform:*

(50) $$l_{m,j}^{*}\left(s\right)=s{\left[F^{-1}\left(s\right)w\left(s\right)\right]}_{mM+j}.$$

Utilizing the final-value theorem with (50), we acquire the final outcome.

**Corollary 4.**  *The stationary rejection rate is*

(51) $$L=\lim_{s\to 0+}s^{2}{\left[F^{-1}\left(s\right)w\left(s\right)\right]}_{1}.$$

## 6. Sample Calculations

In the numeric calculations, four rejection probability functions are used:

(52) $$\begin{array}{c} d_{1}\left(i\right)=\left\{\begin{array}{ccc} 0, & \mathrm{if} & i<10,\\ {\left(\frac{i-15}{15}+\frac{1}{3}\right)}^{5} & \mathrm{if} & 10\leq i<25,\\ 1, & \mathrm{if} & i\geq 25, \end{array}\right. \end{array}$$

(53) $$\begin{array}{c} d_{2}\left(i\right)=\left\{\begin{array}{ccc} 0, & \mathrm{if} & i<10,\\ \frac{i-15}{15}+\frac{1}{3} & \mathrm{if} & 10\leq i<25,\\ 1, & \mathrm{if} & i\geq 25, \end{array}\right. \end{array}$$

(54) $$\begin{array}{c} d_{3}\left(i\right)=\left\{\begin{array}{ccc} 0, & \mathrm{if} & i<10,\\ \frac{{\left(i-10\right)}^{3}}{375} & \mathrm{if} & 10\leq i<15,\\ \frac{i-15}{15}+\frac{1}{3} & \mathrm{if} & 15\leq i<20,\\ -\frac{{\left(25-i\right)}^{3}}{375}+1 & \mathrm{if} & 20\leq i<25,\\ 1, & \mathrm{if} & i\geq 25, \end{array}\right. \end{array}$$

(55) $$\begin{array}{c} d_{4}\left(i\right)=\left\{\begin{array}{ccc} 0, & \mathrm{if} & i<10,\\ 1.005\left(1-e^{-\frac{i-10}{3}}\right), & \mathrm{if} & 10\leq i<25,\\ 1, & \mathrm{if} & i\geq 25. \end{array}\right. \end{array}$$

**Figure 1:**
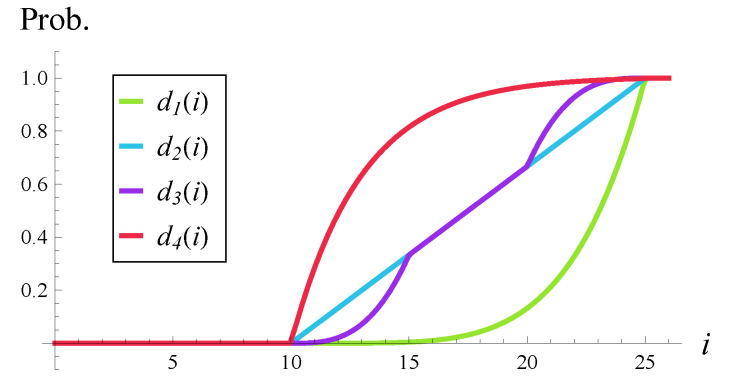
Rejection probabilities used in Section 6.

Functions $d_{1}$–$d_{4}$ are depicted in Figure 1. As we see, in its operating range i∈[10,25), function $d_{1}$ is polynomial, function $d_{2}$ is linear, function $d_{4}$ is exponential, and function $d_{3}$ is of mixed shape.

These types of rejection probability functions were proposed in the literature. Specifically, a linear function was suggested in [10], a polynomial function of higher degree was recommended in [12,13], an exponential function was used in [15], while the mixed shape, exactly like $d_{3}$, was proposed in [18].

Note that we have $d_{1}\left(i\right)\leq d_{2}\left(i\right)\leq d_{4}\left(i\right)$, which means that $d_{2}$ is more forceful than $d_{1}$, while $d_{4}$ is even more forceful than $d_{2}$, in terms of rejection probability. As for $d_{2}$ and $d_{3}$, neither of them is more forceful than the other in general; they operate differently in different intervals.

The service process matrices are as follows:

(56) $$L_{1}=\left[\begin{array}{ccc} 2.210292 & 0.000425 & 0.004774\\ 0.008853 & 0.010528 & 0.005395\\ 0.010978 & 0.456375 & 0.000890 \end{array}\right],$$

(57) $$L_{0}=\left(L^{*}+L_{1}\right)/p-L_{1},$$

where p∈(0,1] is a parameter, and

(58) $$L^{*}=\left[\begin{array}{ccc} -2.234245 & 0.003871 & 0.014883\\ 0.006713 & -0.485407 & 0.453918\\ 0.003075 & 0.002403 & -0.473721 \end{array}\right].$$

Matrices $L_{0}$ and $L_{1}$ are crafted in such a manner that, regardless of *p*, the service rate is always μ=1. Parameter *p* in (57) has no special meaning. It is only used to obtain arbitrary values of the correlation coefficient, which can be accomplished using Formula (4). In the remainder, three values of *p* are used: p=0.0213, for which (4) gives R=0.05 (weak correlation); p=0.0991, for which it gives R=0.2 (moderate correlation); and p=1, for which (4) gives R=0.5 (strong correlation).

Finally, the waiting room capacity N=25 is assumed.

The interarrival distribution is

(59) $$G\left(t\right)=1-0.6e^{-3t}-0.4e^{-t/2},$$

which has the expected value of 1 and a variance of 2.33. Therefore, the arrival rate equals 1, but the arrival process is significantly non-Poisson. The hyper-exponential distribution used in (59) is very useful in practice due to its simplicity and the fact that, through proper parameterization, we can obtain an arbitrarily large variance without altering the arrival rate.

The system load equals ρ=1, if not specified otherwise. This is modified in Section 6.3 only, where the impact of load on the rejection process is investigated.

### 6.1. Impact of Rejection Probabilities

**Figure 2:**
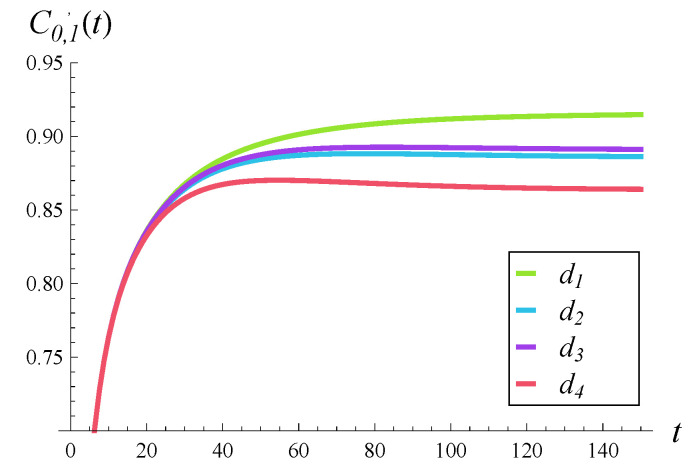
Intensity of departure process in time for rejection probabilities $d_{1}$–$d_{4}$ and weakly correlated service processes (R=0.05).

**Figure 3:**
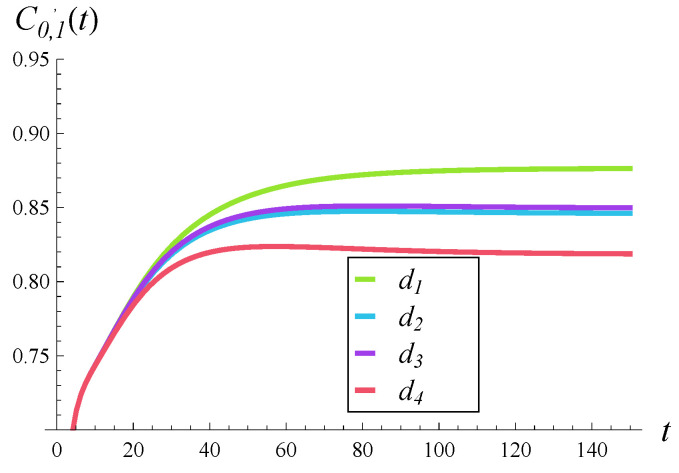
Intensity of departure process in time for rejection probabilities $d_{1}$–$d_{4}$ and moderately correlated service processes (R=0.2).

**Figure 4:**
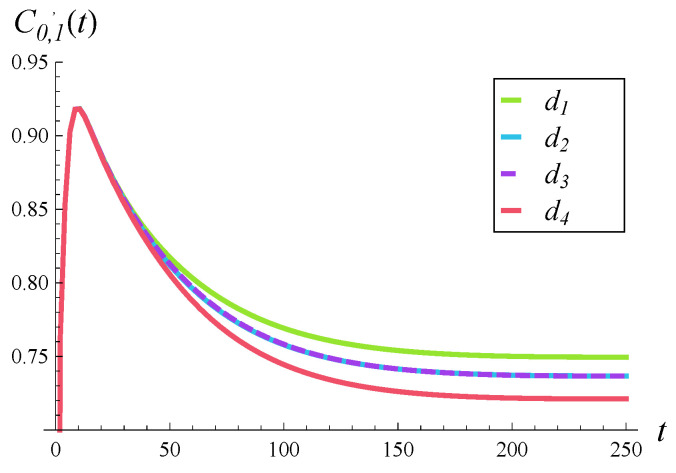
Intensity of departure process in time for rejection probabilities $d_{1}$–$d_{4}$ and a strongly correlated service process (R=0.5).

In Figure 2, Figure 3 and Figure 4, the impact of function d(i) on the departure process is depicted. Specifically, in each of these figures, the time-dependent intensity of the departure process is shown for functions $d_{1}$–$d_{4}$. (The color scheme is the same as in Figure 1, i.e., the green curve represents the departure intensity for function $d_{1}$.)

Figure 2, Figure 3 and Figure 4 differ in that a different correlation strength was assumed when each of them was generated. Specifically, Figure 2 was obtained for a weak correlation of the service process (R=0.05), Figure 3 for a moderate correlation (R=0.2), and Figure 4 for a strong correlation (R=0.5).

The first thing we notice is that the more forceful function d(i) is, the lower the intensities of the departure process are over the entire time axis. This could have been expected. When comparing the $d_{2}$ and $d_{3}$ curves, we can see that they are very close to each other, but $d_{3}$ produces slightly greater departure intensities. Perhaps this is due to the fact that $d_{3}\left(i\right)\leq d_{2}\left(i\right)$ for low values of *i* (i≤15). In other words, it is more important which of the two is less forceful for low queue sizes.

Now, when we compare the effect of shape of function d(i) for different correlations in Figure 2, Figure 3 and Figure 4, a very surprising phenomenon can be seen. Namely, the greatest impact of d(i) is observed neither for weak nor strong, but rather for moderate, correlations. The distance between the green and red curves is clearly the greatest in Figure 3, where the correlation is moderate. It is smaller when the correlation is weak (Figure 2) and even smaller when the correlation is strong (Figure 4).

This effect can perhaps be explained as follows. When the correlation is weak, the queue sizes are often small and all functions $d_{1}$–$d_{4}$ operate in the range where they are zero, i.e., i≤10. This causes relatively small differences between departure intensities. When the correlation is strong, the queue sizes are often very high, near 25, so all functions $d_{1}$–$d_{4}$ operate in the range where they are close to 1. This again causes relatively small differences between departure intensities. When the correlation is moderate, the queue sizes are moderate as well, so all functions $d_{1}$–$d_{4}$ often operate somewhere between i=10 and i=25, where they differ from each other the most. This causes relatively large differences between departure intensities.

As for the convergence time to the stationary departure rate, wecan see that it is similar for both weak and moderate correlations (Figure 2 and Figure 3)—it takes about 100–120 s before the departure intensity becomes stable. In the case of strong correlation in Figure 4, it takes about twice as long before the departure intensity stabilizes. The shape of function d(i) appears to have a much smaller impact than the correlation on the convergence time to the stationary regime.

### 6.2. Impact of Correlation

**Figure 5:**
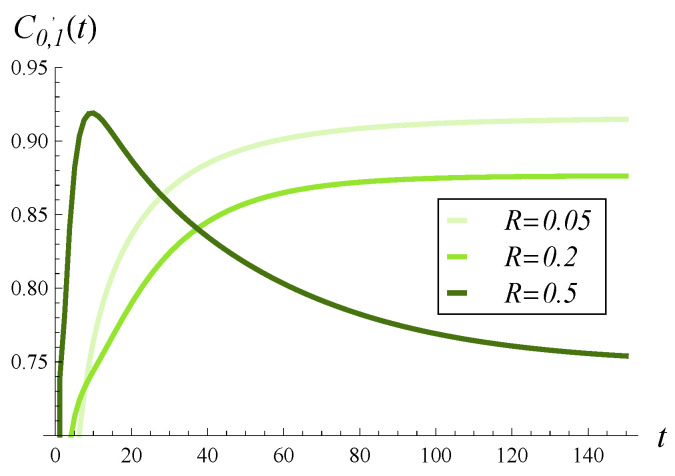
Intensity of departure process in time for three levels of correlation and polynomial rejection probabilities, $d_{1}$.

**Figure 6:**
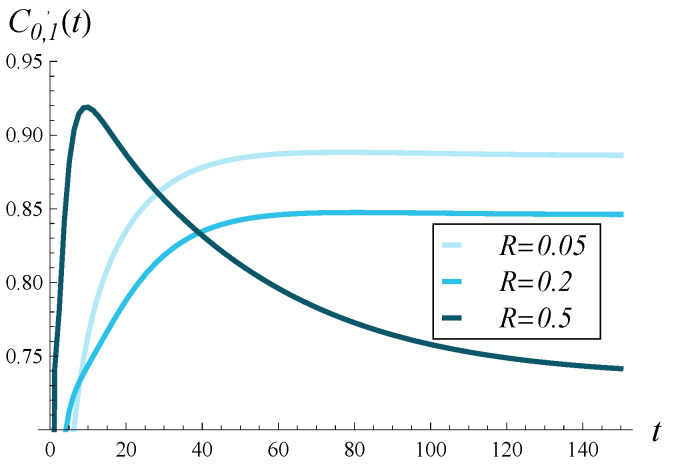
Intensity of departure process in time for three levels of correlation and linear rejection probabilities, $d_{2}$.

**Figure 7:**
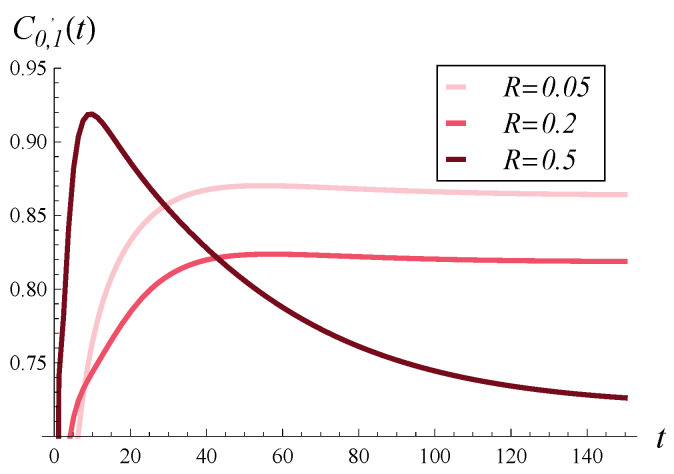
Intensity of departure process in time for three levels of correlation and exponential rejection probabilities, $d_{4}$.

In Figure 5, Figure 6 and Figure 7, the direct influence of the correlation strength on the departure process is shown. Namely, in all of these figures, the time-dependent intensity of the departure process is depicted for R=0.05, R=0.2, and R=0.5. Figure 5, Figure 6 and Figure 7 differ in that a different function d(i) was assumed in each of them. Specifically, Figure 5 was obtained for the $d_{1}$ function, Figure 6 for the $d_{2}$ function, and Figure 7 for $d_{4}$. The graph for function $d_{3}$ is omitted, as it is almost identical to that obtained for $d_{2}$ (Figure 6).

In these figures, we first notice that increasing correlation causes a deterioration in the departure intensity in the stationary regime, regardless of d(i). However, in the transient case, the picture is less clear. Shortly after the start, the departure intensity for R=0.5 is greater than for R=0.2 and R=0.05 (again, regardless of d(i)). Similarly, very shortly after the start, the departure intensity for R=0.2 is greater than for R=0.05.

Secondly, the influence of correlation on the departure intensity is generally much stronger than that of function d(i). There are much greater differences between the curves in Figure 5, Figure 6 and Figure 7 than for those in Figure 2, Figure 3 and Figure 4.

### 6.3. Impact of Load

In this section, we check the impact of system load on the job rejection rate. To vary the load, we use the following service process matrices:

(60) $$L_{1}^{\prime }=L_{1}/\rho ,$$

(61) $$L_{0}^{\prime }=\left(\left(L^{*}+L_{1}\right)/p-L_{1}\right)/\rho ,$$

where $L_{1}$ and $L^{*}$ are given in (56) and (58), respectively. Parameter *p* in (61) has exactly the same function as in (57)—it allows for the correlation strength of the service process to be set without changing the service rate, μ. Parameter ρ in (60) and (61) enables the service rate to be adjusted. Specifically, we have μ=1/ρ, regardless of *p*. As the average interarrival time is 1 (according to (59)), parameter ρ in (60) and (61) is therefore equal to the system load, defined in (3).

**Figure 8:**
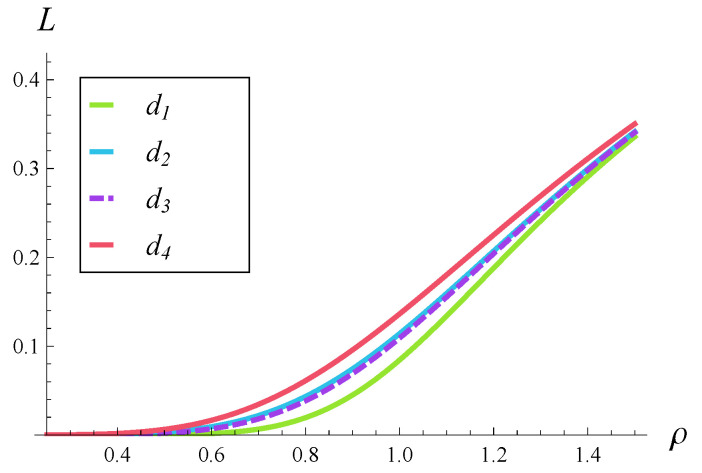
Rejection rate vs. load for rejection probabilities $d_{1}$–$d_{4}$ and weakly correlated service process (R=0.05).

**Figure 9:**
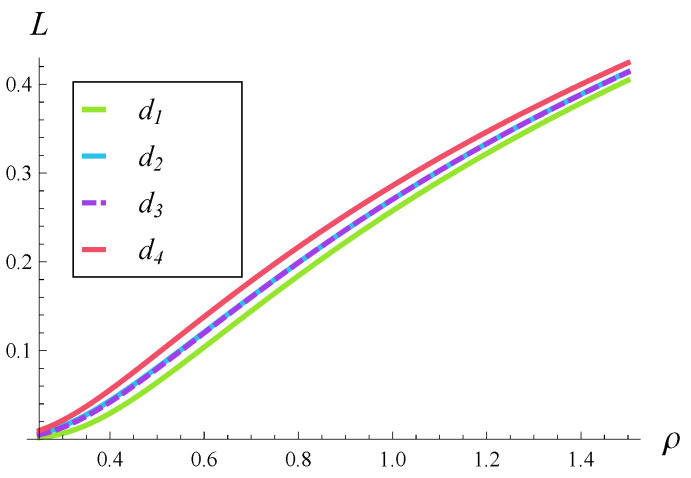
Rejection rate vs. load for rejection probabilities $d_{1}$–$d_{4}$ and strongly correlated service process (R=0.5).

In Figure 8 and Figure 9, the stationary rejection rate versus the load is depicted for functions $d_{1}$–$d_{4}$. Figure 8 differs from Figure 9 in that Figure 8 was generated for a weak correlation, R=0.05, while Figure 9 was generated for a strong correlation, R=0.5.

As we can see in Figure 8 and Figure 9, the system load has a substantial influence on the stationary rejection rate. The effect of the shape of d(i) is minor—all the curves are close to each other in both Figure 8 and Figure 9, regardless of the correlation.

**Figure 10:**
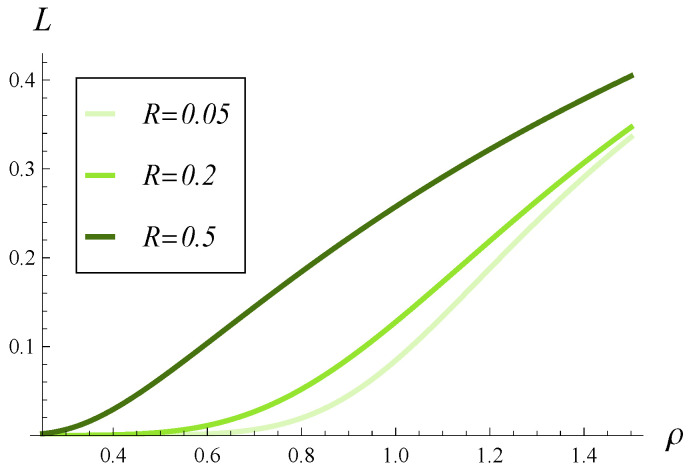
Rejection rate vs. load for three levels of correlation and polynomial rejection probabilities, $d_{1}$.

**Figure 11:**
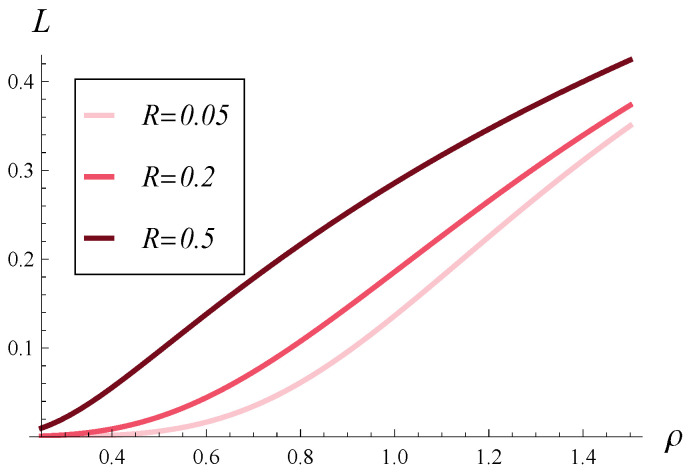
Rejection rate vs. load for three levels of correlation and exponential rejection probabilities, $d_{4}$.

In Figure 10 and Figure 11, the stationary rejection rate versus the load is depicted for correlations of 0.05, 0.2, and 0.5. Figure 10 was generated for function $d_{1}$ while Figure 11 was generated for function $d_{4}$.

As we can see, the effect of correlation on the stationary rejection rate is much stronger than the effect of d(i) function. Indeed, the spread between the curves within Figure 10 and Figure 11 is much greater than that within Figure 8 and Figure 9.

Furthermore, Figure 10 and Figure 11 show that a strong correlation can cause a very high rejection rate, even if the system is underloaded. For instance, if ρ=0.6 and $d_{4}$ functions are applied, the stationary rejection rate is as high as L=0.138. If the ρ=0.6 and $d_{1}$ functions are used, the stationary rejection rate is still very high, L=0.104.

**Figure 12:**
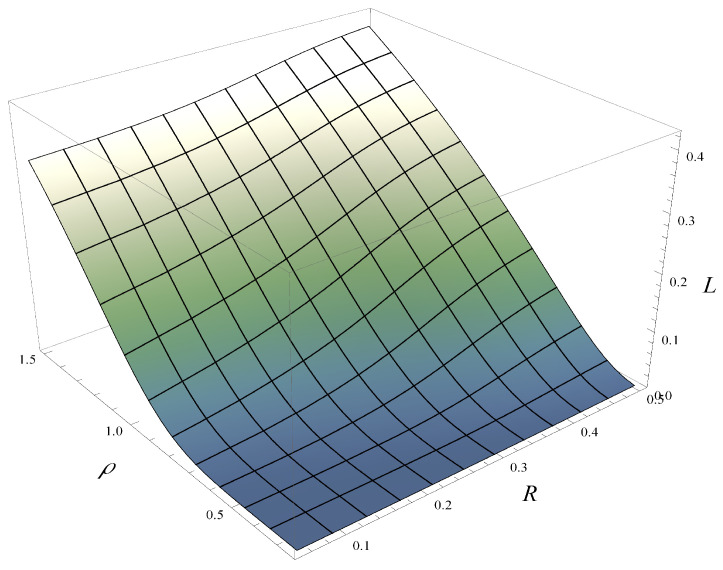
Rejection rate vs. correlation and load for polynomial rejection probabilities, $d_{1}$.

**Figure 13:**
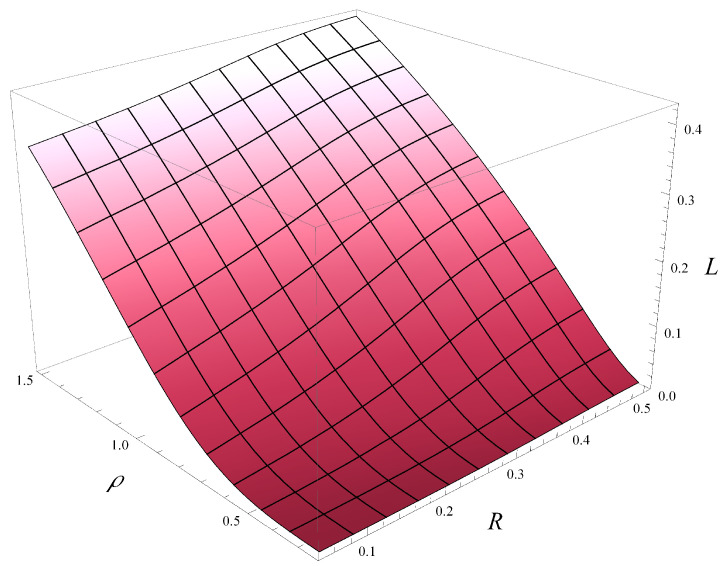
Rejection rate vs. correlation and load for exponential rejection probabilities, $d_{4}$.

Finally, in Figure 12 and Figure 13, the combined impact of correlation and load on the stationary rejection rate is depicted. Figure 12 was obtained for function $d_{1}$, while Figure 13 was obtained for function $d_{4}$.

As seen in Figure 12 and Figure 13, both the correlation strength and the system load have a strong effect on the rejection rate. However, the influence of the correlation is slightly less pronounced—this can be observed by comparing the gradients along the ρ and *R* axes. In this context, the impact of function d(i) is far less significant—Figure 12 and Figure 13 do not differ from each other very much.

### 6.4. Simulation Results

In this section, we compare the simulation results obtained using OMNeT++ simulator [42] with the theoretical results. This is, naturally, meant to ensure that the derivations are error-free.

To accomplish that, the queueing model specified in Section 3 was faithfully implemented in OMNeT++ and parameterized according to (52)–(59). In total, 12 separate simulation scenarios were executed, with different combinations of rejection probabilities and correlation coefficients. In each scenario, 30 million simulated jobs arrived at the system, and the departure rate was observed.

The simulation outcomes are gathered in the last column of Table 1. They can be matched with the theoretical outcomes gathered in the second column. We can see that the difference between the theoretical and simulated results is very small in every scenario.

## 7. Conclusions

In this study, a thorough analysis of the departure and rejection processes of a very general queueing model was carried out. The considered model incorporates passive and active queue management, an arbitrary correlation of the sizes of arriving jobs—resulting in a correlation with service times—and an arbitrary distribution of interarrival times. It was argued that these features of the model are not artificial—they are known to occur in computer networking and many other real-world realizations of queueing systems.

The theoretical contributions of the paper consist of formulae for the expected number of jobs departing within a specified time interval, the intensity of the departure process at an arbitrary time, the stationary departure rate, the expected number of jobs rejected within a specified interval, the rejection intensity at an arbitrary time, and the rejection rate.

In numerical examples, the impact of correlation, rejection probabilities d(i), and system load on the departure and rejection processes was presented, revealing both expected and unexpected phenomena. As shown, the stationary departure rate decreases with increasing correlation strength and rejection probabilities, which could have been expected. In the transient regime, however, a counterintuitive effect can be observed: the intensity of the departure process can be temporarily higher in the instant of a strong correlation than in the instance of weak correlation.

Another interesting fact is that the greatest impact of rejection probabilities was observed neither for weak nor strong, but rather for moderate, correlations.

In general, the impact of correlation on the departure process was much greater than that of the shape of function d(i), while the impact of system load was even greater than that of correlation. Moreover, a strong correlation increased the convergence time to the stationary regime.

## Figures and Tables

**Table 1:** Theoretical and simulated departure rate, *C*, for various system parameterizations.

Parameters	Theoretical *C*	Simulated *C*
$d_{1}$, R=0.05	0.9156	0.9155
$d_{2}$, R=0.05	0.8859	0.8863
$d_{3}$, R=0.05	0.8908	0.8908
$d_{4}$, R=0.05	0.8636	0.8635
$d_{1}$, R=0.2	0.8723	0.8724
$d_{2}$, R=0.2	0.8416	0.8421
$d_{3}$, R=0.2	0.8453	0.8454
$d_{4}$, R=0.2	0.8142	0.8143
$d_{1}$, R=0.5	0.7424	0.7415
$d_{2}$, R=0.5	0.7296	0.7289
$d_{3}$, R=0.5	0.7296	0.7299
$d_{4}$, R=0.5	0.7141	0.7140

## Data Availability

Data is contained within the article.
